# Draft Genome Sequence of *Mycobacterium elephantis* Strain Lipa

**DOI:** 10.1128/genomeA.00691-15

**Published:** 2015-06-25

**Authors:** Alexander L. Greninger, Gail Cunningham, Joanna M. Yu, Elaine D. Hsu, Charles Y. Chiu, Steve Miller

**Affiliations:** Department of Laboratory Medicine, UCSF, San Francisco, California, USA

## Abstract

We report the draft genome sequence of *Mycobacterium elephantis* strain Lipa from a sputum sample of a patient with pulmonary disease. This is the first draft genome sequence of *M. elephantis*, a rapidly growing mycobacterium.

## GENOME ANNOUNCEMENT

*Mycobacterium elephantis* is a rapidly growing mycobacterium, originally isolated from a lung abscess in an elephant in 2000, that has been occasionally isolated from human clinical samples, most commonly sputum specimens (1–3). *M. elephantis* has also been reported to have been isolated from a cervical lymph node, although its relation to clinical disease is unclear (2). The notable phenotypic characteristics of *M. elephantis* include a relatively slow growth rate for a rapid growing mycobacterium, smooth colonies producing a pale yellow pigment with age, and ability to grow on 5% NaCl in Lowenstein-Jensen medium, along with a unique high-performance liquid chromatography (HPLC) mycolic acid profile (3).

Rapidly growing mycobacteria constitute a commonly isolated population of acid-fast bacillus in the clinical microbiology lab of varying clinical importance (4, 5). We sequenced the first draft genome of *M. elephantis* from a sputum sample of a patient in 2003 with pulmonary disease. The isolate was originally typed as *M. elephantis* based on partial 16S sequencing.

DNA from *M. elephantis* strain Lipa was extracted using the Qiagen EZ1 kit, and paired-end libraries were prepared using the Nextera XT DNA library kit followed by sequencing on an Illumina MiSeq instrument. Sequences were adapter and quality (Q20) trimmed using Cutadapt, *de novo* assembled using SPAdes v3.5, metagenomically screened for contaminating sequence with SURPI, and annotated via Prokka v1.1 (6–9). A total of 9,000,614 paired-end reads of average length 115 nucleotides were recovered after trimming. *De novo* assembly yielded 234 contigs for a total assembly size of 5,187,616 bp with an *N*_50_ of 42,430 bp, an average coverage of 196×, and a total of 5,022 coding sequences. Contiguity was most likely disrupted by the high G+C content (68%) along with several high-copy-number integrases, transposases, and recombinases that were longer than sequence read length. Other high-copy-number contigs included those containing genes to *mlaE* phospholipid ABC transporter permease, for which *M. elephantis* strain Lipa had 19 different homologs in the genome.

The five closest BLASTN hits to the complete 16S sequence from the isolate were other *M. elephantis* 16S sequences with 99.4 to 100% identity. The Lipa strain contained three putative beta-glucosidase genes. By Comprehensive Antibiotic Resistance Database analysis, the Lipa strain includes an *arr1* rifampin ADP-ribosyl transferase (85% by amino acid to *M. vanbaalenii* PYR-1) and a *blaF* beta-lactamase (10).

### Nucleotide sequence accession numbers.

This whole-genome shotgun project has been deposited at DDBJ/ENA/GenBank under the accession no. LBNO00000000. The assembly described in this paper is the first version, LBNO01000000.

## References

[1] Shojaei H, Magee JG, Freeman R, Yates M, Horadagoda NU, Goodfellow M 2000 *Mycobacterium elephantis* sp. nov., a rapidly growing non-chromogenic mycobacterium isolated from an elephant. Int J Syst Evol Microbiol 50:1817–1820. 11034492 10.1099/00207713-50-5-1817

[2] Turenne C, Chedore P, Wolfe J, Jamieson F, May K, Kabani A 2002 Phenotypic and molecular characterization of clinical isolates of *Mycobacterium elephantis* from human specimens. J Clin Microbiol 40:1230–1236. doi:10.1128/JCM.40.4.1230-1236.2002. 11923337 PMC140400

[3] Tortoli E, Rindi L, Bartoloni A, Garzelli C, Mantella A, Mazzarelli G, Piccoli P, Scarparo C 2003 *Mycobacterium elephantis*: not an exceptional finding in clinical specimens. Eur J Clin Microbiol Infect Dis 22:427–430. doi:10.1007/s10096-003-0950-2. 12827531

[4] Greninger AL, Langelier C, Cunningham G, Keh C, Melgar M, Chiu CY, Miller S 29 4 2015 Two rapidly growing mycobacterial species isolated from a brain abscess: first whole genome sequence of *Mycobacterium immunogenum* and *Mycobacterium llatzerense*. J Clin Microbiol. doi:10.1128/JCM.00402-15. PMC4473206 25926490

[5] De Groote MA, Huitt G 2006 Infections due to rapidly growing mycobacteria. Clin Infect Dis 42:1756–1763. doi:10.1086/504381. 16705584

[6] Seemann T 2014 Prokka: rapid prokaryotic genome annotation. Bioinformatics 30:2068–2069. doi:10.1093/bioinformatics/btu153. 24642063

[7] Naccache SN, Federman S, Veeraraghavan N, Zaharia M, Lee D, Samayoa E, Bouquet J, Greninger AL, Luk K-C, Enge B, Wadford DA, Messenger SL, Genrich GL, Pellegrino K, Grard G, Leroy E, Schneider BS, Fair JN, Martínez MA, Isa P, Crump JA, DeRisi JL, Sittler T, Hackett J, Miller S, Chiu CY 2014 A cloud-compatible bioinformatics pipeline for ultrarapid pathogen identification from next-generation sequencing of clinical samples. Genome Res 24:1180–1192. doi:10.1101/gr.171934.113. 24899342 PMC4079973

[8] Martin M 2011 Cutadapt removes adapter sequences from high-throughput sequencing reads. EMBnet.journal 17:10–12. doi:10.14806/ej.17.1.200.

[9] Bankevich A, Nurk S, Antipov D, Gurevich AA, Dvorkin M, Kulikov AS, Lesin VM, Nikolenko SI, Pham S, Prjibelski AD, Pyshkin AV, Sirotkin AV, Vyahhi N, Tesler G, Alekseyev MA, Pevzner PA 2012 SPAdes: a new genome assembly algorithm and its applications to single-cell sequencing. J Comput Biol 19:455–477. doi:10.1089/cmb.2012.0021. 22506599 PMC3342519

[10] McArthur AG, Waglechner N, Nizam F, Yan A, Azad MA, Baylay AJ, Bhullar K, Canova MJ, De Pascale G, Ejim L, Kalan L, King AM, Koteva K, Morar M, Mulvey MR, O’Brien JS, Pawlowski AC, Piddock LJ, Spanogiannopoulos P, Sutherland AD, Tang I, Taylor PL, Thaker M, Wang W, Yan M, Yu T, Wright GD 2013 The comprehensive antibiotic resistance database. Antimicrob Agents Chemother 57:3348–3357. doi:10.1128/AAC.00419-13. 23650175 PMC3697360

